# A Neural Network Model for Color Element Data Analysis for Urban Spatial Environment

**DOI:** 10.1155/2022/4674620

**Published:** 2022-08-21

**Authors:** Xiaotang Xia, Tingyang Li

**Affiliations:** ^1^School of Urban Construction, Wuhan University of Science and Technology, Wuhan, Hubei 430070, China; ^2^Wuhan Business University, Wuhan, Hubei 430000, China

## Abstract

In this paper, a CNN model for color element data analysis of the urban spatial environment is constructed through an in-depth study of color element data analysis. This paper investigates a high-order structure formed by a few nodes; it proposes a motif-based graph autoencoder MODEL, combining redefined first- and second-order similarities and perfectly integrating motif structure and autoencoder. The algorithm first proposes an efficient graph transformation method to add the influence of central nodes. It then offers a primary awareness mechanism to aggregate the information of noncentral neighbors. Cen GCN_D and Cen GCN_E outperform the latest algorithms in node classification, link prediction, node clustering, and network visualization. As the number of network layers increases, the advantages of these two variants become progressively more prominent. This paper uses a support vector machine to implement classification validation based on CNN. The experimental results show that when 450 images are randomly selected as training data, the classification accuracy obtained by using the features of different CNN output layers is distributed between 91.4% and 95.2%. When the training set of the experiment reaches more than 300, the accuracy can exceed 90%, and the experimental results corresponding to different training sets a more stable trend. Finally, the trained classifier model is obtained in this thesis, which achieves the purpose of fast classification prediction based on CNN for color element data analysis of urban spatial environments.

## 1. Introduction

With the continuous development of cities and the gradual speeding up of the urbanization process, the urban color landscape in the city is getting more and more attention, and urban color design has become a hot topic in urban planning and construction. Urban color design is an incomparably complex system engineering, including urban design, urban planning, architecture, landscape, management, color science, and other related theoretical knowledge; designers need to consider the role and influence of various factors in the city, combined with the city color management department, together to put forward urban color design direction and goals [1]. The urban color design aims to shape the characteristics of an urban color image, maintain the ideal urban landscape, build a design system with different control categories and degrees, and put the design content into practice. Since the 1990s, with the prominence of urban color contradictions, research institutions have gradually started research and related practical work for urban color to solve the current urban color problems, which has played a specific positive effect on the harmony, control, and management of the urban color landscape [2]. However, most cities (including those that have completed urban color design studies) are overwhelmed by the modern buildings and materials rising from the ground and are unable to form a coordinated, harmonious, and unified urban color landscape [3]. The failure and low operability of urban color control is a common and core issue that must be addressed in the current urban color design approach.

From the perspective of human visual cognition, the urban color element environment can be regarded as an environment with spatial cognitive benefit, spatial construction auxiliary shape, and spatial repair reconciliation and has a perceptual emotional information transfer function that simple geometric forms do not have, and therefore it becomes one of the indispensable elements in the process of the urban landscape and spatial landscape construction, with an irreplaceable and essential role, highlighting the urban population. It also plays an irreplaceable and critical role in the construction of urban landscape and spatial landscape, highlighting the universal value pursuit and historical heritage of urban people [4]. In the era of traditional urban architecture, the material properties of the building, the level of technology, and the social, political, and economic conditions in which it is located have all imposed constraints on the use of urban color and formed the color application habits of urban architecture with individual characteristics and rich regional style, thus making the color landscape in the city present more harmonious and unified style characteristics [5]. With the level of science and technology in human society, the technical barriers to constructing modern urban space have become smaller and smaller. The new construction materials and cross-regional exchange of convenient, easy-to-achieve separation of architectural color and building materials significantly reduce the regional restrictions on selecting materials in the construction of urban space to shape the diverse urban color landscape [6]. It provides more practical conditions for shaping various urban color and landscape features and opens the door to the problem of urban color abuse.

Deep learning has made significant breakthroughs recently, providing new ideas to solve old and contemporary urban and rural planning problems. The technology represented by CNN has also made breakthroughs in color analysis and has been widely used in natural language processing [7]. Compared with traditional machine learning algorithms, CNN eliminates many preprocessing processes and performs much better processing audio, image, video, and other data. At the same time, deep learning has been a hot research topic in academia. New network architectures are emerging every year, and the effectiveness of deployment and implementation for various tasks is gradually improving [8]. For users, well-packaged deep learning libraries have steadily lowered the threshold of using deep learning technology. Using deep learning libraries, users can easily choose the matching network models according to data requirements and deploy and manage their deep learning networks. In the era of big data, the large and diverse data contain various helpful information that further drives academic research and business development [9]. In network data, each sample is no longer an isolated entity; there are multiple relationships between them. Neural network algorithms do not take advantage of the higher-order structural information inherently present in the network. Thus, these algorithms do not fully mine the network structure, resulting in many missing relationships. In the context of big data, networks become more complex, diverse, and large-scale. The similarity between shallow pairs of nodes can no longer adequately characterize the structure of existing networks [10]. Therefore, there is an urgent need for warp networks to incorporate more complex higher-order systems in the network and explore more relationships between nodes. In this paper, we will study the neural network model for color element data analysis of the urban spatial environment to improve the algorithm's performance in relevant applications.

## 2. Related Works

With the rapid development of big data technology, combining the protection and research of traditional culture with big data technology is a big trend. There are many studies based on big data technology for traditional culture, and they have got good results [11]. Color is the most intuitive and prominent feature that can distinguish things' surface characteristics. There are many methods of color extraction, including the color histogram method. The color histogram method reflects the proportion of color information of different images in the image color space and is an effective way to research color information. At the same time, the color histogram is based on other coordinate systems and color spaces [11]. It can be used to study color and spatial information by calculating RGB, CIEL*∗a∗b*, HSV, etc. The color aggregation vector method proposed by Nasiri et al. [12] mainly solves the problem that the color moment method and histogram method cannot express the spatial location of image colors clearly. This method is an evolution of the color histogram method; the core of the aggregation vector method is dividing the pixels belonging to each histogram bin into aggregated and nonaggregated pixels [12]. Eslami et al. [13] proposed that the purpose of color metrics is to represent the colors seen by everyday people objectively and numerically related to the requirements of a specific application. The concepts of color gamut and color space emerged. The commonly used color metric models in urban color research are closely related to the Munsell color representation system and the CIE color representation system [13]. In color planning, the HIS and HSV models under the Mimsell specimen system and the parallelized representation of RGB models based on primary color mixing are more commonly used [14]. Through these methods, we can digitally integrate and summarize colors and store the results in the form of text, maps, and diagrams in the database as necessary data support for planning.

From the 1990s to the present, research and practical work on urban color have been gradually carried out, and the color landscape of cities has been improved to a certain extent. In recent years, academic research on urban color design theory and practice has gradually expanded from the initial few scholars to an enormous scope. For a city, the more urgent, the more critical, and the more stringent control needs to be solved, and with the fastest results, the color issues we consider the most basic need to be solved [15]. This is because, from the planning manager's point of view, they often expect maximum results with minimal investment in the shortest possible time. At the same time, refinement can be carried out gradually at a later date. Urban color design's most urgent and primary task is to protect, restore, and remedy color pollution in the city's possible historic districts and the coordinated blocks around cultural preservation units [16]. Relatively higher and better pursuits, such as creating a unique “color” of the city and creating a harmonious color atmosphere, are the icing on the cake. After that, it belongs to the overall city color design type, hoping to use the city color design to coordinate and control the city's color landscape. Accordingly, the current types of urban color design can be divided into three major categories: historical and cultural heritage and regional preservation, new urban areas and particular areas of urban color practice, and overall urban color design. Yang H et al. used computer vision technology to construct suitable convolutional neural networks (CNN) to identify the street green view rate, street functional attributes, and pedestrian attributes in public space and made a preliminary exploration of quantitative research on general urban areas based on big visual data [17]. Gu et al. [18] also proposed a model for urban imagery research based on web-based image data; constructed a research framework with urban imagery element composition, dominant direction, characteristic degree, and similarity of urban imagery as modules; and conducted an empirical study for 24 major cities [18].

Due to the significant differences in urban planning and construction patterns, foreign urban color design experiences and patterns cannot be directly copied and applied to Chinese urban color practice [16]. Regarding the practice and research of urban color design, based on foreign color theories and methods, we have been exploring ideas and techniques adapted to the current situation of cities from the beginning stage. We have developed a set of current urban color design research systems. The study of urban spatial environment identification mainly studies urban characteristics, urban planning, and urban design for shaping. So far, the more popular urban design theories and methods in the West focus on the functional design of public space, private space, and shared space from the urban environment landscape and logo design elements [17]. Since the foreign urban construction and urban planning system can reflect the will and vision of designers and planners more, in shaping the overall urban image, it can reflect the personality of the creator of the urban image as well the nature of the urban image more. Therefore, the urban spatial environment identification can often be reflected through urban planning and construction.

## 3. Neural Network Model Based on Data Analysis Construction

CNN is a mixture of convolutional operations and deep neural networks. Convolutional neural networks are often used for data feature selection and image processing applications because of their symbolic learning capability [19]. Compared to other multilayer network structures, convolutional neural networks have more powerful abilities; neurons are the basic units that make up a neural network. A neural network contains multiple structural layers, and each layer contains various neurons. In the map, the vectors *x*_1_, *x*_2_, and *x*_3_ are the input signals to the neurons, and +1 represents their bias, usually represented by the symbol *b*. The neuron is described as follows:

(1) $$\begin{array}{c} f_{\left(w^{t}-x\right)}=\sum_{i=1}\frac{\sqrt{w_{i}x_{i}-b}}{w_{i}-x_{i}}, \end{array}$$

where *x* represents the input to the neuron, W and *b* are the neuron's weight term and bias term, respectively, and the function *f* is a nonlinear activation function that represents the response to the input signal. The input signal is the output after a nonlinear operation through a specific activation function of a single neuron, which is calculated as follows:

(2) $$\begin{array}{c} h_{w-b}^{x}=\sum \frac{w^{t}-1}{f\left(w^{t}-x\right)}. \end{array}$$

Convolutional neural networks have a unique structure with a hierarchy consisting of an input layer, an output layer, and single or multiple hidden layers. *L*1 is the input layer, while *x*_1_, *x*_2_, and *x*_3_ are the input signals and +1 bias terms. The middle of the network below is the hidden layer, while *L*2 is the final output layer used to output the final result. In this case, the network layers are interrelated, and the previous layer's output is used as input to the next layer and propagated backward. The vector dimensions of the information and hidden layers are usually set according to the problem under study, while the parameters of the hidden layers are determined experimentally. The neurons in each layer of the convolutional network receive the information output from a set of units in the previous layer and perform a series of convolutional operations. Usually, the computation of the convolutional layers can be represented by the following equation:

(3) $$\begin{array}{c} x_{i}=\sum x_{i}^{l+1}+w_{ij}-b\sqrt{{\left(l+j\right)}_{ie}^{mj}}. \end{array}$$

In equation (3), *l* represents the number of convolutional layers. In contrast, *w* means the convolutional kernel, *b* is the bias term of the network, and *Mj* is denoted as the input feature image. Since the convolutional layers have the feature of weight sharing, the parameters will be significantly reduced. Now, suppose there is a model with 1000 hidden layer nodes and an input image with a pixel size of 1000 × 1000, while if a fully connected approach is used, the number of parameters will be 1000 × 1000 × 1000 = 109; while if 100 convolutional kernels are used for processing, and the size ofconvolutional kernels is 10 × 10, there will be 10 × 10 × 100 = 104 parameters, and it can be seen that the convolutional neural network makes a significant reduction in the number of parameters.

A general convolutional neural network can be broadly divided into three layers: an input layer, a hidden layer, and an output layer. Since neural networks are generally used to perform computer vision tasks, the input layer often accepts three-dimensional or one-dimensional arrays, that is, three-channel RGB images or single-channel grayscale images. In addition, the input layer is used to normalize the input data from the [0, 255] pixel value interval to the [0, 1] interval to improve the learning efficiency of the network since the network training generally requires learning and parameter updating using the gradient descent method. The output layer is used to output the network results, and the output of the trained network is the final fitted result; for the network to be prepared, the difference between the predicted output and the natural result is calculated by the loss function during training, so that the weights in the previous layers can be updated by back propagation to make the network fit the result more in line with the demand [20]. This process is called the network learning process. The hidden layer is the most critical layer of a convolutional neural network and can be divided into three unique layers: a convolutional layer, a pooling layer, and a fully connected layer. A hidden layer can contain one or more convolutional and pooling layers and generally includes a fully connected layer. The input data can be abstracted by repeatedly performing convolution and pooling operations. This layer-by-layer abstraction represents the features of the input data at different degrees and scales. Therefore, this class of layers is the most critical layer for feature extraction and is the fundamental reason for the powerful performance of convolutional neural networks. Since the fully connected layer can be replaced by a convolutional layer of the same size as the input image, only the convolutional and pooling layers will be described in detail.

### 3.1. Convolutional Layer

A deep convolutional neural network's hidden layer generally contains many convolutional layers, which convolve the input image and then output the result and use it as the input of the next layer for forwarding propagation. The convolution operation can extract the deep features of the abstracted image data, and these removed features can make the final prediction result of the network more accurate. Hence, the convolution layer is the essential core layer of the convolutional neural network. Each convolutional kernel corresponds to a bias term in convolutional neural networks. After convolving each input, this bias term needs to be added and activated by the activation function, and the final result is the output of this convolutional layer. Adding a bias term makes the network fitting result more accurate, and the network convergence speeds faster. Therefore, the convolutional computation process after adding the bias term is as follows:

(4) $$\begin{array}{c} c_{i}^{j}=\sum_{m=1}w_{m,n}\times a_{m-i}+a_{n-j}-b, \end{array}$$

where *c* is the convolution result, *k* is the convolution kernel size, *w* is the convolution kernel weight, *a* is the input image to be convolved, and *b* is the bias term. In the actual calculation, two other critical parameters for the convolution operation, namely, stride and padding, impact the output data size after convolution. The stride represents the distance that the convolution kernel slides on the original image data during each convolution, and the setting of this parameter can control the abstraction level of the output image after convolution; the more significant the stride is, the larger the abstraction level is, and the smaller the image size is. Generally, the step size is set to 2, which means that the convolution kernel extracts feature from every region of the image; the fill setting is related to the size of the convolution kernel, which is generally divided by two and rounded upward as the size of the image boundary fill area. The fill mode is usually chosen as zero fill. Therefore, for a step size of *s*, a filling of *p*, an input image size of *w∗w∗*4, a convolutional kernel size of *k*, and several *m*, the output image size is as follows:

(5) $$\begin{array}{c} {\text{out}}_{w}^{k}=\sum \left[\frac{w+k+m}{\sqrt{s-1}}-2p\right]\times \left[\frac{w-k-m}{\sqrt{s+1}}-2p\right]. \end{array}$$

### 3.2. Pooling Layer

For most convolutional neural networks, there is often a pooling layer after each convolutional layer, and the role of pooling layer is mainly to abstract the input data further and also to perform feature dimensionality reduction to reduce the model parameters and improve the operation speed and prevent overfitting to a certain extent. Pooling can be divided into average pooling and maximum pooling, and their calculation formulas are expressed as follows:

(6) $$\begin{array}{c} b_{\left(i-j\right)}=\sum_{n=1}\frac{\sqrt{a_{i-m}+a_{j-n}}}{n_{h}-n_{w}}\times \left(h-w\right),\\ b_{\left(i+j\right)}=\sum \frac{a_{i-m}-a_{j+n}}{\max\left(a_{i-m}+a_{j+n}\right)}. \end{array}$$

Pooling is also not essential for convolutional neural networks. For example, a 2*∗*2 pooling operation can be replaced by a convolution with a step size of 2; thus, reducing the number of operations in a pooling layer. However, this approach is not stable and may give better results on sizeable deep network structures but may give worse results on some small networks. A convolutional neural network is a multilayer structure containing a feature extractor consisting of several convolutional layers, subsampling layers (pooling layers), and a connection layer in a convolutional layer; a neuron is connected to only some of its neighboring neurons. A convolutional layer in a CNN usually contains several feature maps, each consisting of several rectangularly arranged neurons, and the neurons in the same feature map share weights. The shared consequences are the convolutional kernel.

## 4. Color Element Data Analysis Model Construction for Urban Spatial Environment

**Figure 1:**
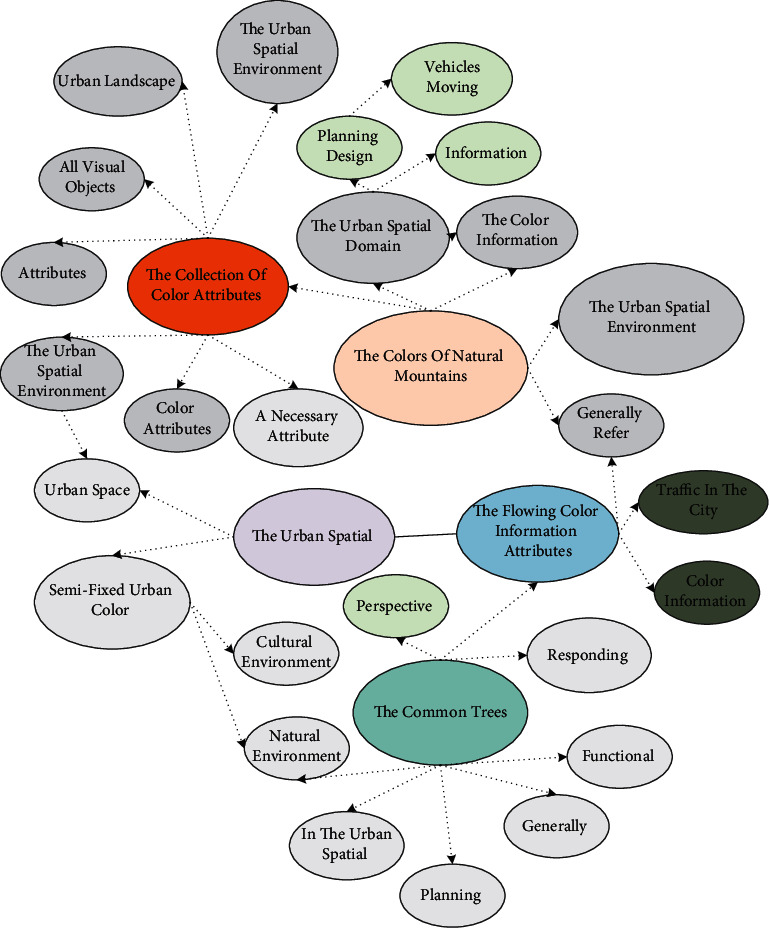
Urban color planning corresponds to the system process.

Color is a necessary attribute of the urban spatial environment, and no urban landscape can exist independently without its color attributes. Urban color, in the general sense, can be regarded as the collection of color attributes of all visual objects in the urban spatial environment. It includes the color attributes of buildings and structures in the city, the colors of natural mountains and geography as the background of the urban spatial domain, and even the people and vehicles moving in the urban spatial entity [21]. From the perspective of color creation and planning design of the urban spatial environment, we often divide the color information covered in the urban spatial environment into fixed color information attributes, semifixed color information attributes, and mobile color information attributes. The selected color information attributes refer to the relatively slow change of color attributes and their carriers, such as the urban spatial environment accounted for a relatively large number of buildings and structures belonging to the ranks of fixed color information attributes; and semifixed urban color information attributes refer to the urban spatial environment that requires a certain amount of time before the more significant changes in color attributes and their carriers, such as the common trees in the city landscape gardening and street landscape vignettes, flowing. The flowing color information attributes usually refer to the users of urban space entities and generally refer to the harmonious color attributes and their carriers, such as the flow of people and traffic in the city. The urban color planning covered in this thesis only refers to fixed and semifixed color information attributes in the urban spatial environment. Color planning mainly reflects the color characteristics and differences embodied in the different functional divisions of regional cities, which can be attributed to the overall positioning of specific city colors: responding to the impact of changes in the regional natural environment, cultural environment, and artificial built environment, as well as the color evolution and adaptation caused by the expansion of urban space, urban industrial structure adjustment, and the strengthening of spatial and temporal links between cities in the regional development environment. The matching is shown in Figure 1.

According to the composition mechanism of color landscape elements in the urban space environment, the color attributes can be divided into natural environment color and artificial environment color. Natural environment color refers to the natural color elements and their carriers, such as the sky, water system, and mountains, which are within reach of our visual perception. The artificial environment color refers to the color elements and their carriers reflected by the artificially constructed structures, including the systems and rigid pavements used daily by urban residents; all belong to the artificial environment color category. In the specific color planning and design implementation, we can divide the urban space environment color into background color, primary color, auxiliary color, and embellishment color. Background color refers to the natural geographic environment on which the urban spatial environment is based.

In contrast, the primary color refers to the main color information presented on the facade of the buildings in the urban spatial entity. The additional and embellishment colors are the color information elements that account for less on the buildings and play a supporting role or decoration. Through the color classification of the urban space environment, we can see that, from different perspectives, different urban space color environments can be divided; these color elements, based on differences but also have a specific internal connection, constitute the urban space we know color landscape style.

**Figure 2:**
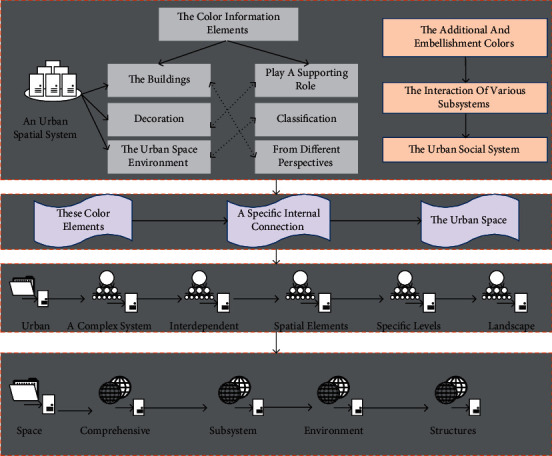
Urban spatial data analysis process.

An urban spatial environmental system is a complex system composed of interacting and interdependent spatial elements with specific levels, structures, and functions in a particular social environment. It has the following characteristics: first, the urban spatial environment system is an essential subsystem of the extensive urban system, enabling the city to exist in material form as a comprehensive system. Secondly, the urban spatial environment system is the projection of the interaction of various subsystems of the urban social system on the urban land. The composition of modern urban space has become increasingly diversified, incorporating many traditional outdoor public activities into the indoor spatial environment, resulting in the emergence of microurban rooms in integrated buildings. This is a powerful complement to the complete connotation of the urban public space system, which meets the spatial needs of people for modern social life. Thus, it is measured regarding urban space's social and physical attributes. The analysis of urban space data is shown in Figure 2.

**Figure 3:**
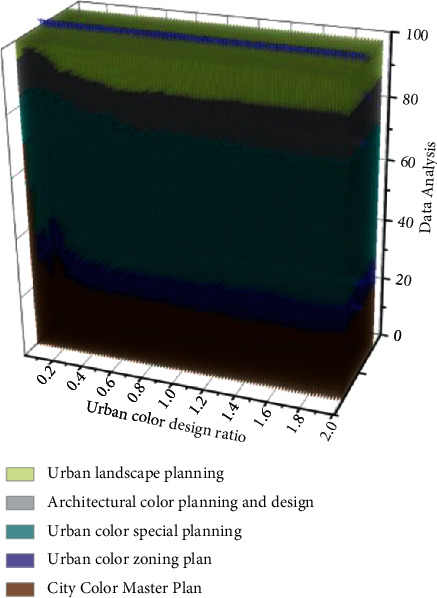
Data analysis of urban color design types.

The urban spatial environment is, first of all, a material space whose core function is to carry out all kinds of social activities in the city and is the primary field for people to cognize and experience the city. The identification system of the urban spatial environment is complex in a particular social setting, composed of spatial identification elements that interact and depend on each other. It has a certain level, structure, and function. The urban spatial environment has many material and nonmaterial aspects [22]. It is necessary to organize and manage them effectively and orderly to make them an integral part of the urban spatial environment identification system. Building a structural model is feasible to contain these complicated elements in an orderly way. The structural model can analyze and categorize the ingredients in many aspects and highlight the characteristics of the urban spatial environment. Therefore, this chapter focuses on the urban spatial environment identification system, composed of the structural model of urban spatial environment identification and the identification of typical forms of urban spatial environment. The establishment and use of the urban spatial environment identification system are essentially the processes of discovering the characteristics of the urban spatial environment and its effective identification. The analysis of urban color design type data is shown in Figure 3.

The spatial environment created according to different design concepts will be different, and there are pronounced differences in characteristics between them, with prominent branding of the concept. Suppose the information on the features of the urban spatial environment that can reflect the history and culture of the city is refined and processed in a certain way. In that case, it can be associated with the corresponding elements so that the purpose of identification can be achieved. For example, suppose the information reflecting the characteristics of the urban spatial environment is refined and processed in a certain way so that it can be associated with the corresponding design concept. In that case, the purpose of identification from the perspective of the design concept can be realized. It is assumed that linear and point-like buildings, streets, and spatial forms are a set of information vectors reflecting a particular design concept in an urban spatial environment, and each information vector has multiple spatial environment characteristics. By assigning each information vector to different categories according to the design concept through fuzzy clustering, a mapping can be established between the information of the urban spatial environment and the characteristics of the design concept signs. Through the analysis and understanding of people's urban imagery, we can grasp the environmental elements and their features that affect people's perception and classify these characteristics to make the urban spatial environment highlight its unique signs for identification. This kind of clustering is based on analyzing and generalizing different people's urban imagery. The title's premise is to evaluate and analyze a certain number of model urban imagery and its corresponding urban elements. The clustering method in the feature clustering identification model not only adopts the fuzzy logic method commonly used at present but also other methods can be used according to the actual situation or application effect. In addition, when the boundary of spatial environment classification is not very obvious, some corresponding preprocessing can be carried out before sorting. For example, some fusion of spatial environment feature information can be used to solve the problem of spatial environment information recognition under uncertain conditions with few paradigms and multiple feature spaces, especially for urban spatial environment imagery feature recognition, which has a good effect.

## 5. Analysis of Results

### 5.1. Neural Network Model Analysis of Color Element Data Analysis of the Urban Spatial Environment

Color harmony is essential in the overall control of the city such as the ratio between adjacent colors between the hue brightness vividness relationship and similar hues between environmental colors, hue harmony, and hue harmony method to adjust the discordant urban color [23]. (1) The contrast relationship between adjacent colors: through the edge contrast of adjacent colors, people can identify subtle color differences, the hue also in this contrast, in the interaction of various visual sensory changes. (2) Environmental color harmony relationship: the color harmony with the form, building materials, and places will change accordingly. 

**Figure 4:**
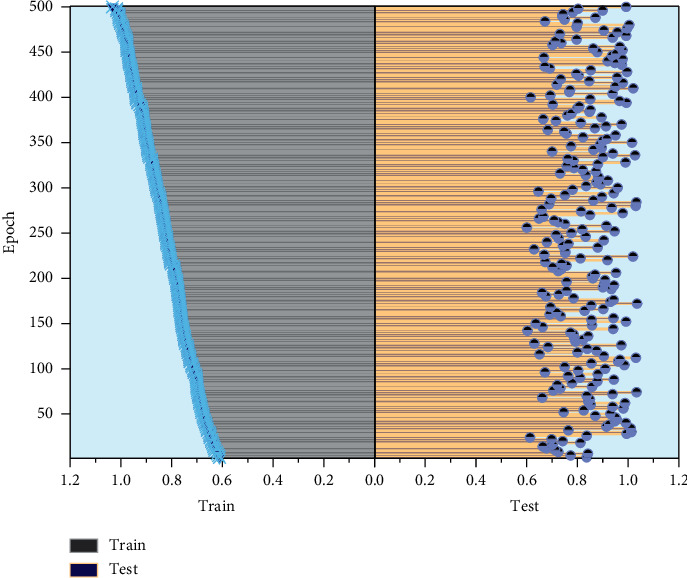
Variation of model accuracy with a period.

This paper divides the primary data set into 90% of the data as the training set and the remaining 10% as the test set during the actual training. For the training parameters, the batch size is set to 32, the initial learning rate is set to 0.002, and the learning rate is multiplied by 0.2 every 30 training rounds for 500 games. The training data set is randomly disordered in each round. The weights of the Adam optimization algorithm were developed to decay from 4 to 10, and the two hyperparameters *β*_1_ and *β*_2_ were set to default values of 0.9 and 0.999, respectively. The variation of model accuracy over time is shown in Figure 4.

**Figure 5:**
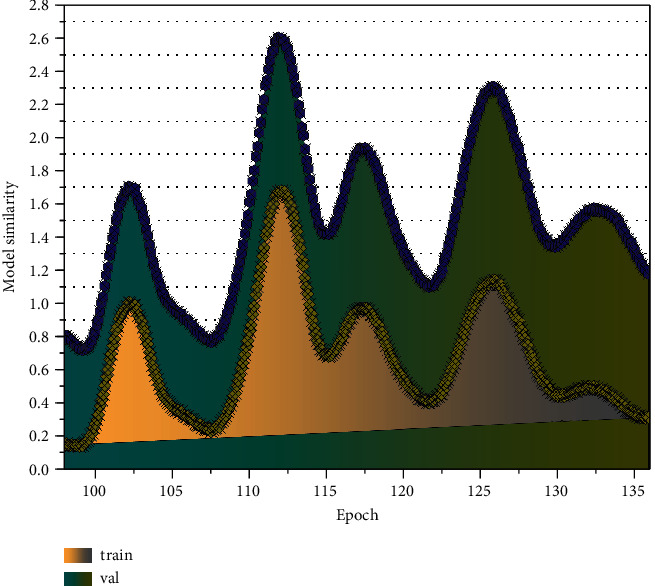
Variation of model similarity with a period.

For convolutional neural networks, a well-designed network structure is a basis for the convergence of the model. At the same time, a suitable data set is needed to ensure whether the network can accomplish the set learning goals and whether the trained network model can meet the final task requirements. A good data set can speed up the convergence of the network so that the network can learn the mapping between the input and the target output faster and complete the task. On the other hand, it can significantly reduce the network's performance, making it unable to learn the correct parameters and thus underfitting or overfitting. This affects the generalization performance of the network and may even cause the network to fail to converge. Once the network meets the accuracy requirements, the web can be used to compute the example data. In the Keras framework, the h5py library was used to load the previously trained U-Net network for prediction, and all the image data of the streetscape were input in batches. After 9 hours and 469955 calculations, the pixel percentages of greenery, sky, buildings, pedestrian streets, and motorized streets in all the streetscape data were finally obtained. At this point, the task of the image segmentation network has been fully completed. The variation of model similarity with a period is shown in Figure 5.

A convolutional neural network was built to analyze and calculate the color elements of the urban spatial environment previously acquired, and the results of the classification of all color elements were obtained in a short time. The softmax function is used in the category, and its calculation formula is as follows:

(7) $$\begin{array}{c} \text{Softmax}\left(x-1\right)=\int_{x=1}\frac{e^{x_{i-1}}-1}{\sqrt{e^{x_{1}}+e^{x_{2}}}}. \end{array}$$

Equation (7) indicates here that it is one of two categories, in this case, 1 or 2, so the network outputs a matrix with two values, one for the prepredicted color value and the other for the postpredicted color value. It can be easily deduced that the sum of the two output values is 1. Since the two predicted values are reciprocal, when evaluating the color element scores, it is only necessary to focus on using the cheerful color expected value. Based on the abovementioned principles, it is possible to quantify the color element data for all urban spatial environments.

### 5.2. Color Element Data Analysis Realization of the Urban Spatial Environment

**Figure 6:**
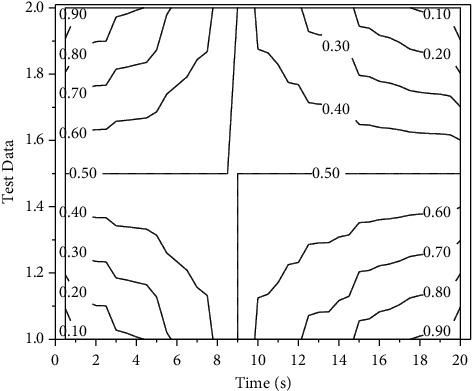
Visualization of the training set distribution.

The natural colors in the urban space environment are influenced by the city's geographic location, topography and terrain, natural vegetation, and other factors, presenting different visual perceptions. Natural colors are also divided into constant and nonconstant colors. Land, mountains, rivers, and other natural colors that can remain stable over a long period are standard colors with a particular law of change; nonconstant colors are represented by the sky, vegetation, and urban neon, which have the characteristics of changing with time. The natural colors are in people's daily lives and affect society's physical and mental feelings. Artificial colors in urban color, as the name suggests, are artificially planned and designed in urban advertisements, streets, public vehicles, equipment and facilities, and pedestrian clothing colors. City streets, high-rise buildings, unique buildings, and other relatively fixed colors; pedestrian clothing, cars, and other colors constitute the flow of color; city advertising, lighting, street decorations, signs, and other colors form a temporary color. Artificial colors accompany people's daily work and life, profoundly influence the surrounding environment, and harmonize with natural dyes to jointly determine the color appearance of the city. The results obtained by SVM classification based on the existing CNN model are better; there are about 477 training data. To verify the effect of data size on the experimental results, fc8 is selected as the output layer, and the size of the experimental data will be adjusted to get the corresponding classification results. The number of all data sets is 716, of which 0.1, 0.2, 0.3, 0.4, 0.5, 0.6, 0.7, 0.8, and 0.9 are selected as training data, and the others are used as test data for experiments. The visualized image of the training set is shown in Figure 6.

**Figure 7:**
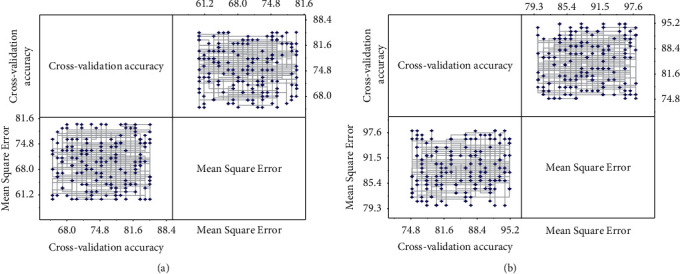
Experimental results of different training sets.

In each selection of training data, the entire data set will be randomly disrupted to ensure the fairness of the data. Still, because there is a specific error in each data selection, this thesis only uses the experimental data as a reference to analyze the overall change trend. For example, the cross-validation accuracy obtained when the number of training data was 358 is 94.8%. The result is relatively higher than the experimental results when the number of training samples was 430, 502, and 574, but this does not indicate that the experimental results corresponding to the number of pieces of training were 368 are better. The results must be analyzed based on the overall trend of change. As can be seen, the minimum number of training data is 72, the maximum number is 716, and the accuracy rate is guaranteed to be above 80%. Also when the amount of data reaches about 300, the overall trend is more stable. When the data come to 300 or more, the mean square error of the trained SVM model is not high, and it can be guaranteed to be around 0.1, which indicates that the experimental results are more reliable. The experimental results of different training sets are shown in Figure 7. The experimental data based on mass spectral images contain 149 images in the most categories and 53 shots in the least. The amount of data in all types of the network databases far exceeds the experimental data of mass spectral images, corresponding to the better classification results obtained from the experiments. Therefore, it can be concluded that the data analysis neural network model applied in this paper has a good recognition ability and is suitable for designing color elements in urban spatial environments.

## 6. Conclusion

The generation and development of the urban spatial environment is a selfgrowing process with a long-time dimension. As an essential element affecting the urban landscape, the buildings in the urban spatial environment have a relatively long-term existence; that is to say, their influence on the urban landscape is relatively long from the date of their construction to the date of their demolition. Therefore, renovation and renewal, which only change the color of the building's appearance instead of demolishing the building itself, become an essential means of shaping and improving the urban landscape economically and operably. This paper proposes a convolutional neural network algorithm, Cen GCN, on a scale-free network. This paper presents a graph transformation method to capture the influence of the central node on its surrounding neighbors. The power of the central node on similar neighbors increases, and the impact on dissimilar neighbors decreases. This paper also proposes a significant awareness to aggregate the information of noncentral node neighbors. In experiments, this paper presents two variants, Cen GCN_D and Cen GCN_E. Both variants outperform the latest convolutional neural network algorithms in node classification, link prediction, node clustering, and network visualization. Even though the scale-free nature is based on degree features, significant results can still be achieved using eigenvalue centrality. This study shows that convolutional neural networks can be improved by considering central nodes and centrality. It is also demonstrated that the introduction of node centrality can deepen the convolutional neural network. When the number of neural network layers reaches ten, the algorithm still achieves long-lasting results. The neural network based on the AlexNet model has high feature learning performance, and the output fully connected layers can all represent the information contained in the mass spectral images relatively well; thus, obtaining high accuracy and low mean square error. In particular, the last fully connected layer, fc8, receives higher classification accuracy of 95.2% than other fully connected layers. The inherent neural network model based on millions of training images does not have high requirements in terms of training set size and has a relatively stable recognition capability when the data volume reaches 300 or more. In this paper, the trained classifier model is obtained to classify and predict the color element data analysis of the urban spatial environment based on CNN.

## Data Availability

The data used to support the findings of this study are available from the corresponding author upon request.

## References

[1] Law S., Seresinhe C. I., Shen Y., Gutierrez-Roig M. (2020). Street-Frontage-Net: urban image classification using deep convolutional neural networks. *International Journal of Geographical Information Science*.

[2] Moayedi H., Moatamediyan A., Nguyen H., Bui X. N., Bui D. T., Rashid A. SA. (2020). Prediction of ultimate bearing capacity through various novel evolutionary and neural network models. *Engineering with Computers*.

[3] Jahani A., Allahverdi S., Saffariha M., Alitavoli A., Ghiyasi S. (2022). Environmental modeling of landscape aesthetic value in natural urban parks using artificial neural network technique. *Modeling Earth Systems and Environment*.

[4] Cui Z., Henrickson K., Ke R., Wang Y. (2020). Traffic graph convolutional recurrent neural network: a deep learning framework for network-scale traffic learning and forecasting. *IEEE Transactions on Intelligent Transportation Systems*.

[5] Yang X., Li X., Ye Y., Lau R. Y. K., Zhang X., Huang X. (2019). Road detection and centerline extraction via deep recurrent convolutional neural network U-Net. *IEEE Transactions on Geoscience and Remote Sensing*.

[6] Allam Z. (2019). Achieving neuroplasticity in artificial neural networks through smart cities. *Smart Cities*.

[7] Majd R. D., Momeni M., Moallem P. (2019). Transferable object-based framework based on deep convolutional neural networks for building extraction. *Ieee Journal of Selected Topics in Applied Earth Observations and Remote Sensing*.

[8] Hang R., Liu Q., Hong D., Ghamisi P. (2019). Cascaded recurrent neural networks for hyperspectral image classification. *IEEE Transactions on Geoscience and Remote Sensing*.

[9] Zhou L., Zhang S., Yu J., Chen X. (2020). Spatial–temporal deep tensor neural networks for large-scale urban network speed prediction. *IEEE Transactions on Intelligent Transportation Systems*.

[10] Zhao L., Song Y., Zhang C. (2020). T-GCN: a temporal graph convolutional network for traffic prediction. *IEEE Transactions on Intelligent Transportation Systems*.

[11] Zeng W., Lin C., Lin J. (2021). Revisiting the modifiable areal unit problem in deep traffic prediction with visual analytics. *IEEE Transactions on Visualization and Computer Graphics*.

[12] Nasiri V., Darvishsefat A. A., Rafiee R., Shirvany A., Hemat M. A. (2019). Land use change modeling through an integrated Multi-Layer Perceptron Neural Network and Markov chain analysis (case study: arasbaran region, Iran). *Journal of Forestry Research*.

[13] Eslami E., Salman A. K., Choi Y., Sayeed A., Lops Y. (2020). A data ensemble approach for real-time air quality forecasting using extremely randomized trees and deep neural networks. *Neural Computing & Applications*.

[14] Saralioglu E., Gungor O. (2022). Semantic segmentation of land cover from high resolution multispectral satellite images by spectral-spatial convolutional neural network. *Geocarto International*.

[15] Chen Y., Zhu K., Zhu L., He X., Ghamisi P., Benediktsson J. A. (2019). Automatic design of convolutional neural network for hyperspectral image classification. *IEEE Transactions on Geoscience and Remote Sensing*.

[16] Hou X., Wang K., Zhong C., Wei Z. (2021). ST-trader: a spatial-temporal deep neural network for modeling stock market movement. *IEEE/CAA Journal of Automatica Sinica*.

[17] Yang H., Yu B., Luo J., Chen F. (2019). Semantic segmentation of high spatial resolution images with deep neural networks. *GIScience and Remote Sensing*.

[18] Gu K., Zhou Y., Sun H., Zhao L., Liu S. (2020). Prediction of air quality in Shenzhen based on neural network algorithm. *Neural Computing & Applications*.

[19] Xie J., He N., Fang L., Plaza A. (2019). Scale-free convolutional neural network for remote sensing scene classification. *IEEE Transactions on Geoscience and Remote Sensing*.

[20] Gogikar P., Tyagi B., Gorai A. K. (2019). Seasonal prediction of particulate matter over the steel city of India using neural network models. *Modeling Earth Systems and Environment*.

[21] Sun R., Tu Z., Fan L. (2020). The correlation analyses of bacterial community composition and spatial factors between freshwater and sediment in Poyang Lake wetland by using artificial neural network (ANN) modeling. *Brazilian Journal of Microbiology*.

[22] Zorzi S., Maset E., Fusiello A., Crosilla F. (2019). Full-waveform airborne LiDAR data classification using convolutional neural networks. *IEEE Transactions on Geoscience and Remote Sensing*.

[23] Ebhota V. C., Isabona J., Srivastava V. M. (2019). Environment-adaptation based hybrid neural network predictor for signal propagation loss prediction in cluttered and open urban microcells. *Wireless Personal Communications*.

